# Polyacrylamide Ferrogels with Ni Nanowires

**DOI:** 10.3390/ma12162582

**Published:** 2019-08-13

**Authors:** Alexander P. Safronov, Bethanie J. H. Stadler, Joseph Um, Mohammad Reza Zamani Kouhpanji, Javier Alonso Masa, Andrey G. Galyas, Galina V. Kurlyandskaya

**Affiliations:** 1 Institute of Natural Sciences and Mathematics, Ural Federal University, 620002 Ekaterinburg, Russia; 2 Institute of Electrophysics, Ural Division RAS, 620016 Ekaterinburg, Russia; 3 Electrical and Computer Engineering, University of Minnesota, Minneapolis, MN 55455, USA; 4 Chemical Engineering and Materials Science, University of Minnesota, Minneapolis, MN 55455, USA; 5 Department of CITIMAC, University of Cantabria, 39005 Santander, Spain; 6 Departamento Electricidad y Electrónica, Universidad del País Vasco UPV-EHU, 48080 Bilbao, Spain

**Keywords:** nickel nanowires, electrochemical deposition, ferrogels, nanomagnetism

## Abstract

Nickel magnetic nanowires (NWs) have attracted significant attention due to their unique properties, which are useful for basic studies and technological applications, for example in biomedicine. Their structure and magnetic properties were systematically studied in the recent years. In this work, Ni NWs with high aspect ratios (length/diameter ~250) were fabricated by electrodeposition into commercial anodic aluminum oxide templates. The templates were then etched and the NWs were suspended in water, where their hydrodynamic size was evaluated by dynamic light scattering. The magnetic response of these NWs as a function of an external magnetic field indicates a dominant shape anisotropy with propagation of the vortex domain wall as the main magnetization reversal process. The suspension of Ni NWs was used in the synthesis of two types of polyacrylamide ferrogels (FGs) by free radical polymerization, with weight fractions of Ni NWs in FGs of 0.036% and 0.169%. The FGs were reasonably homogeneous. The magnetic response of these FGs (hysteresis loops) indicated that the NWs are randomly oriented inside the FG, and their magnetic response remains stable after embedding.

## 1. Introduction

Low-dimensional magnetic materials based on pure transition metals have attracted special attention due to both their unique properties and expected applications in high-density magnetic recording, spintronics, microwave circuits, magnetic sensors, catalysts, soft actuators, and biomedicine [1,2]. One-dimensional nickel nanowires (NWs) are very promising magnetic materials, whose physical and chemical properties depend on their size and aspect ratio [3,4]. Well-established electrochemical deposition methods have used porous anodic aluminum oxide (AAO) templates [2,5,6] to fabricate iron oxide ellipsoids/spindles or nickel NWs with different geometrical parameters [7]. Efficient control of both the crystal growth and the geometry of the NWs can make possible the development of NWs with tuned magnetic properties, opening the door for new and interesting applications. For example, properties of long Ni NWs having a high aspect ratio (>100) may have advantages for high-density magnetic recording and biomedical applications [8,9].

Composite materials consisting of magnetic nanoparticles, nanorods, or nanowires embedded into a hydrogel matrix are called ferrogels (FGs) [10,11]. Because they are sensitive to the application of a magnetic field, FGs have attracted special attention for novel applications in regenerative medicine and magnetic biosensing, i.e., areas of research where their ability to mimic basic properties of natural tissues are useful [12,13]. Although there have been several studies of FGs containing spherical metallic nanoparticles, there are only a few studies available on nanowires embedded into a hydrogel matrix [7,14,15]. In addition, although there are advanced techniques to evaluate structural properties of solid materials, including composites containing nanoparticles or nanorods and NWs [16,17,18], methods for evaluating the structure of gels and FGs are much less developed. Therefore, there is still lots of work to do on this area. 

Ni nanowires is an attractive model system to study the abovementioned aspect. First of all, there are well developed chemical and electrochemical techniques for their fabrication with stable parameters [1,2,19]. The second advantage is a single phase state. As the material is pure, the phase composition is easier to control and their magnetic responses can be compared with well-known values corresponding to the bulk ferromagnets [19].

In this work, high aspect ratio magnetic Ni nanowires were fabricated by the electrodeposition technique using anodic aluminum oxide templates. Then, FGs with ferromagnetic Ni nanowires were synthesized by free radical polymerization of monomeric acrylamide, and their structure and magnetic properties were studied.

## 2. Materials and Methods

### 2.1. Synthesis of Ni Nanowires

Magnetic Ni NWs were synthesized at room temperature by electrodeposition into commercial anodic aluminum oxide (AAO) templates with an 80 nm pore diameter (InRedox LLC, Longmont, CO 80504, USA). More details about the fabrication process have been given before [19]. Prior to the deposition, a 7-nm-thick Ti layer was sputtered onto one side of the AAO as an adhesion layer, followed by a 200-nm-thick Cu layer to act as a working electrode in a standard three-electrode system. A platinum mesh was used as counter electrode. The Ni electrolyte consisted of 300 g/L NiSO_4_·6H_2_O, 45 g/L NiCl_2_·6H_2_O, and 45 g/L H_3_BO_3_. The pH of the electrolyte was adjusted to 4.5 using diluted NaOH. Ni was deposited into the AAO pores at a constant voltage of 0.9 V. An Ag/AgCl glass electrode was used as a reference electrode. The length of the nanowires was controlled by monitoring the deposited charge. After NW growth, the alumina template was dissolved in 1 M NaOH for 2 h in an ultrasonic bath at room temperature. The obtained suspension was concentrated using a Hermle Z383 centrifuge (Gosheim, Germany), and the sediment was washed several times in distilled water via dispersion/sedimentation cycles until the pH value of the supernatant became neutral. The obtained suspension was then used for the synthesis of FGs with embedded Ni NWs. 

### 2.2. Synthesis of FGs with Ni Nanowires

Blank gel without NWs and FGs (FGs) with Ni NWs were synthesized by free radical polymerization of monomeric acrylamide (AAm) (AppliChem, Darmstadt, Germany) in a 2.7 M water solution. N,N′ methylene diacrylamide (MDAA) (Merck, Schuchardt, Germany) was used as a cross-linker in molar concentration to monomer equal to 1:100. Ammonium persulfate (APS) was used as an initiator in 3 mM concentration. AAm, MDAA, and APS were first dissolved in water with the addition of the suspension of Ni NWs in different concentrations to provide FGs with a low and high concentration of NWs. From now on, these FGs will be denoted as FG1 and FG2, respectively. Next, a catalyst—N,N,N′,N′-tetra-ethyl-methylene-di-amine (TEMED, Merck, Schuchardt, Hohenbrunn, Germany) in 5 mM concentration—was added to the mixture and the reaction took place for approximately 10 min at room temperature. After that, FGs were held for 1 h at 70 °C to complete polymerization. Synthesized FGs were extensively washed in distilled water for 2 weeks with daily water renewal in order to remove residues of APS and TEMED, and to reach an equilibrium aqueous swelling.

### 2.3. Methods

The morphology and composition of the NWs before and after embedding them into FGs were evaluated by both optical microscopy and scanning electron microscopy (SEM) with energy dispersive X-ray spectrometry (EDS). The optical microscopy images were taken in water for swollen FGs. The SEM images were taken using a Zeiss NEON 40 EsBCrossBeam SEM (SEMTech Solutions Inc., North Billerica, MA, USA). For the SEM images of the NWs embedded into the gel, a JEOL JSM-640 (20 kV accelerating voltage) microscope was used (JEOL USA Inc., Peabody, MA, USA). The FG samples were cut into pieces, completely dried, and a thin carbon layer of about 20 nm was deposited onto the surface in order to avoid the electric charging [20]. This method was developed in our previous works [20,21]. It allows obtaining information about structural peculiarities of polymer composite materials with oxide or metallic nanoparticles.

The size distribution of Ni NWs in water suspension was studied by dynamic light scattering (DLS) using a Brookhaven ZetaPlus particle size analyzer (Brookhaven Brookhaven Instruments Corp., Holtville, NY, USA). The electrokinetic zeta-potential of the suspensions was measured by electrophoretic light scattering (ELS) using the same analyzer. All the measurements were made at room temperature. 

Thermal gravimetric analysis (TGA) with differential scanning calorimetry (DSC) and simultaneous quadrupole mass-spectrometry (QMS) was carried out using a NETZSCH STA409 thermal analyzer (NETZSCH-Gerätebau GmbH, Selb, Germany) at a heating rate 10 K/min, in order to determine the concentration of Ni NWs in FGs.

The magnetization versus field (M-H) or hysteresis loops of the Ni nanowires were measured using vibrating sample magnetometry (VSM: Faraday magnetometer of laboratory design). For the NWs inside the AAO template, the M-H loops were measured with the nanowires oriented parallel and perpendicular to the magnetic field, respectively. Hysteresis loops were also measured for the FG samples inside a polymer capsule. Gel and FG masses were carefully measured prior to every magnetic measurement. Magnetic contributions of the Ni NWs were afterwards recalculated, considering the gel matrix diamagnetic contribution.

## 3. Results and Discussion

**Figure 1:**
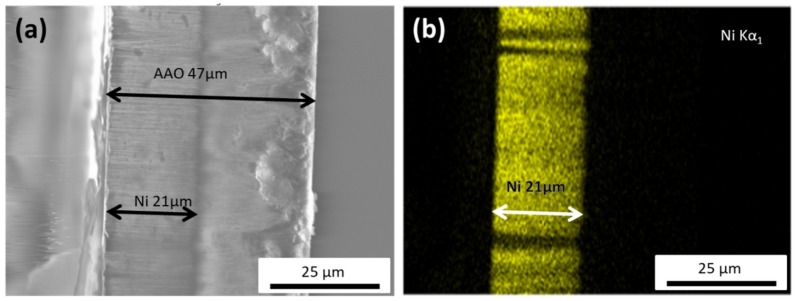
(**a**) Cross-sectional SEM image of the anodic aluminum oxide (AAO) template with the Ni nanowires (NWs) grown inside, indicating a NW’s length of about 21 μm. (**b**) EDS map of the Ni nanowires, showcasing the homogeneous Ni distribution within the nanowire.

As depicted in Figure 1a, the total thickness of the AAO membrane was around 47 μm, while the Ni nanowires length was close to 21 μm. From this SEM image, it is also clearly seen that all the NWs present a uniform length distribution and average diameter of about 80 nm. Figure 1b shows an example of the EDS analysis for the Ni scan. It further confirms the length of the nanowires, and clearly indicates that they are homogeneously composed of pure nickel, noting that the electron beam probes about 1 μm (approximately 10 NWs) into the template.

Although some surface oxidation of the nanowires can still be present, as has been reported before [22], the magnetic responses (the estimated values of the saturation magnetization) indicate that the fabricated material consists of pure Ni NWs.

**Figure 2:**
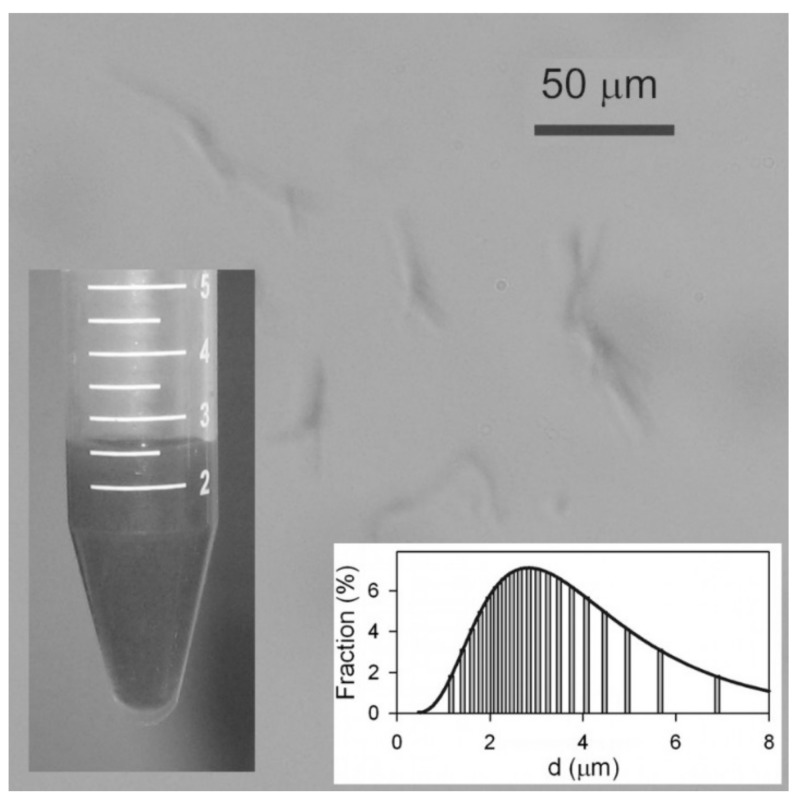
Optical microscopy snapshot of the suspension of Ni NWs in water. Insets: General view of the suspension of Ni nanowires. Apparent particle size distribution in the suspension obtained by DLS.

Analysis of the aqueous dispersion of Ni NWs is presented in Figure 2. Although the concentration of Ni NWs was quite low (below 1% by weight) the suspension was not transparent, but dark gray. Even though the average diameter falls below the resolution of optical microscopy, nevertheless their large lengths made it possible to observe their aggregates with this technique. It is noticeable that NWs in suspension frequently present in small aggregates containing 2–5 NWs, potentially formed during the centrifugation. The suspension was relatively stable—no sedimentation was observed after 1 h. However, in a 24 h period, Ni NWs formed a loose sediment. The limited stability of the suspension has an electrostatic origin.

The electrokinetic zeta-potential of the suspension measured by ELS was found to be −27 ± 2 mV, which means the Ni NWs in the suspension are negatively charged and efficiently repel each other. Most likely, the negative charge on the surface of Ni NWs is the result of their dissolution procedure. Specifically, when the alumina template was dissolved in an alkali, it may yield AlO_2_^−^ anions, which can be adsorbed onto the surface of NWs, thereby providing them a negative electrical charge. Surface oxidation effects could also be contributing to this. It is important to note that the nanowires can be resuspended after sedimentation by a simple shaking procedure (or sonication), which means the NWs experience negligible magnetic attraction to each other.

In addition, DLS measurements were carried out to determine the hydrodynamic size of the NWs in solution. The inset in Figure 2 shows that the particle size distribution (PSD) is wide and it is fitted well by log-normal distribution with a median of 2.8 μm and a logarithmic dispersion of 0.54. The mean value of the apparent hydrodynamic diameter therefore was 3.3 μm. This value is much smaller than the length of the NWs as determined by SEM (21 μm), and can be explained taking into account the basic underlying equation for the evaluation of a particle diameter in DLS, namely the Einstein equation for the diffusion of a sphere in viscous medium:

(1) $$D=\frac{RT}{6\pi \eta r}$$

where *D = D_0_ exp(−Q/kT)* is the diffusion coefficient, *Q* is the activation energy, *T* is the temperature, *k* is the Boltzmann constant, *η* is the viscosity of a liquid, *r* is the radius of the sphere, *R* is the gas constant, and π have its usual meaning. Thus, DLS measurements can provide adequate estimation for the diameters of particles in suspension only if their shapes are close to spherical. Therefore, the obtained mean value (3.3 μm) for the hydrodynamic diameter in the suspension of Ni NWs corresponds to the equivalent spheres, which have the same coefficient of diffusion as measured by DLS in suspension. Meanwhile, certain corrections for the elongated particles can be done.

Thus, the evaluation of the rotational and translational diffusion coefficients for elongated particles based on DLS measurements was reported in Reference [23]. The model by Tirado et al. [24], developed for the hydrodynamic properties of cylinders with a length-to-diameter ratio from 2 up to 30, was elaborated to evaluate the rotational and the translational diffusion coefficients of Ni nanorods in aqueous colloidal dispersion. It was shown that experimental values measured using multiangle DLS fit fairly well the prediction of the Tirado theory for the ensemble of Ni nanorods with an average diameter of 20–40 nm and an average length of 100–250 nm.

We were not able to use the same approach in the present study as the particle size analyzer that we used provided DLS measurements only at 90° scattering and did not allow multiangle scattering, which was necessary for separate evaluation of rotational and translational diffusion coefficients. Besides, the length-to-diameter ratio of the Ni NWs we had studied was substantially larger than that covered by Tirado model. Therefore, assuming that Ni NWs might be considered as very elongated ellipsoidal particles, we used the classical theory of the hydrodynamic flow of ellipsoidal particles developed by Perrin [25]. 

The theory gives a coefficient of the translational mobility of the ellipsoid *M(k)*, which tells by what ratio the mobility and hence the diffusion coefficient of an ellipsoid is less than the mobility (and the diffusion coefficient) of a sphere, whose diameter is equal to the short axis of an ellipsoid, with *k* being the aspect ratio (*k* = *a*/*b*, with *a* being the long axis of the ellipsoid and *b* being the short axis). Coefficient *M(k)* is related to the geometrical factors *G_a_(k)* and *G_b_(k)*, which count for the deviation of an ellipsoid from a sphere with the diameter equal to the long axis (*G_a_(k)*) and equal to the short axis (*G_a_(k)*). *M(k)*, *G_a_(k)*, and *G_b_(k)* are related to the aspect ratio *k* o fan ellipsoid according to the following equations:

(2) $$\begin{array}{l} M(k)=\frac{3}{G_{a}(k)+2G_{b}(k)}\\ G_{a}(k)=\frac{8}{3}{\left[\frac{2k}{1-k^{2}}+\frac{2k^{2}-1}{{\left(k^{2}-1\right)}^{\frac{3}{2}}}\ln\frac{k+\sqrt{k^{2}-1}}{k-\sqrt{k^{2}-1}}\right]}^{-1}\\ G_{b}(k)=\frac{8}{3}{\left[\frac{k}{k^{2}-1}+\frac{2k^{2}-3}{{\left(k^{2}-1\right)}^{\frac{3}{2}}}\ln(k+\sqrt{k^{2}-1})\right]}^{-1} \end{array}$$

Assuming that the shape of Ni NWs can be approximated by ellipsoids, and using the diameter of Ni NWs (80 nm) for the estimation of the short axis, we have calculated the diffusion coefficient of the reference sphere using Equation (1). Then, the ratio of the diffusion coefficient of the reference sphere to the experimental value of the diffusion coefficient, measured by DLS for the suspension of Ni NWs gave the value of the coefficient of translational mobility *M(k)* = 0.0242. Finally, the aspect ratio of the ellipsoid with such translational mobility was calculated using Equation (2), which gave *k* = 240. Such aspect ratio gives 20 μm for the long axis of the ellipsoid. This estimation is fairly close to the observed length of Ni NWs as shown in Figure 1. Thus, if corrected for the ellipsoidal shape of Ni NWs, DLS results are consistent both with the results of optical microscopy and with the SEM images. 

**Figure 3:**
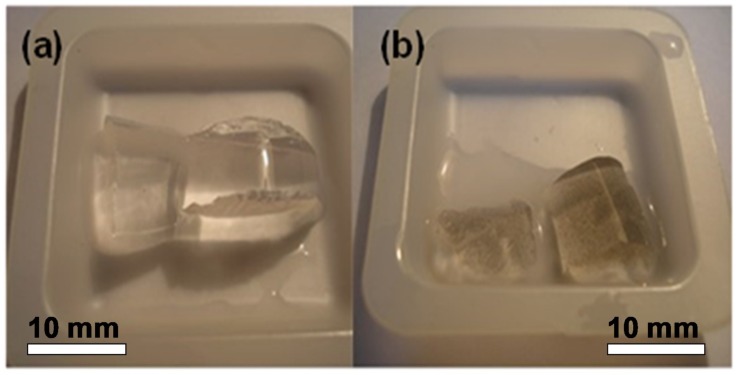
General view of polyacrylamide blank gel (**a**) and ferrogel 2 (FG2) containing a high aspect ratio nickel NWs (**b**).

The equilibrium swelling degree, i.e., the water uptake of FGs was determined as the weight loss of the gel specimen after drying at 70 °C compared to the weight of the dry residue:

(3) $$\alpha =\frac{m-m_{0}}{m_{0}},$$

where *m* is the mass of a swollen gel specimen, and *m*_0_ is the mass of the dry residue. Ni NWs in water suspension were used for the synthesis of two FGs with different concentrations of NWs: FG1 and FG2. In addition to FGs, a blank gel without nanowires was also prepared. Figure 3 shows a general view of gel and FG. The equilibrium swelling degree was found equal to 9.0 in all cases. The obtained materials were reasonably homogeneous at the mesoscopic scale, morphologically stable, and easily cut.

The weight fraction of Ni NWs in swollen FG was determined by thermal gravimetric analysis (TGA). The dry FG1 and FG2 specimens were burned in flowing air by heating up to 1000 °C, and the weight of the residue was measured. Compared to the swollen gel weights, the residue was 0.48% for FG1 and 2.02% for FG2. Assuming that Ni NWs were fully oxidized to Ni oxide during heating, the weight fraction of Ni NWs in dry FGs (ω) was estimated as 0.36% (FG1) and 1.70% (FG2). Using these values, the weight fraction of Ni NWs in swollen FGs was calculated according to the equation:

(4) $$\%\mathrm{Ni}\;\mathrm{NWs}\;=\;\frac{\omega }{1+\alpha }100\%.$$

Thus, the weight fraction of Ni NWs in FG FG1 with low content was 0.036%, and the weight fraction of Ni NWs in FG FG2 with high content was 0.169%.

**Figure 4:**
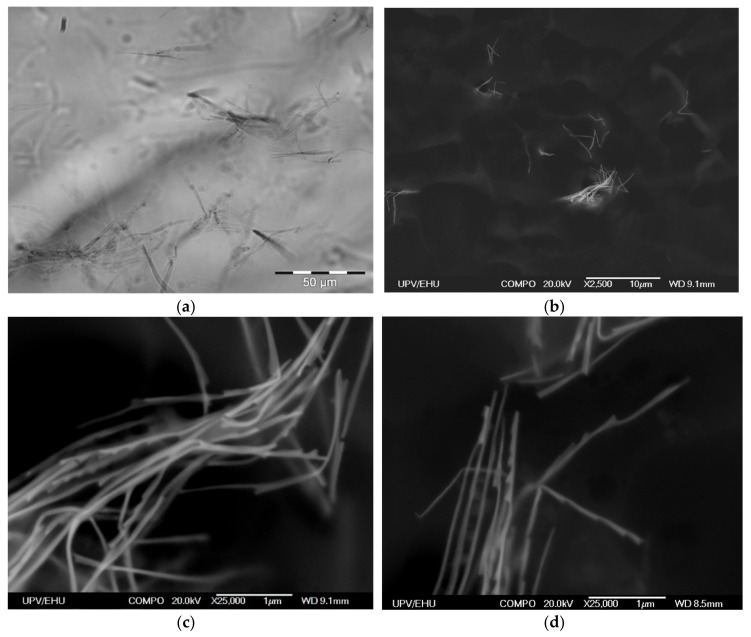
Optical microscopy snapshot of FG1 with embedded Ni NWs (0.036% by weight) (**a**). SEM microphotographs of Ni NWs in dry FG1 from different places and at different magnifications. Weight fraction of NWs is 0.36% by weight (**b**–**d**).

Immobilization of Ni NWs in FG preserves the distribution of NWs from the precursor suspension. Figure 4a presents a typical example of the optical microscopy snapshot of the interior of the FG1 in equilibrium state (without drying). It can be noticed that NWs are randomly arranged both as individual wires and as small aggregates. The volume concentration of NWs is low. Evaluation based on the weight fraction of Ni NWs and densities of Ni and the gel matrix gives 4.9 × 10^−3^% (by volume). This indicates a large separation between different NWs inside the FG structure.

Due to the resolution limitation of the optical microscopy resolution, the internal structure of NWs aggregates in FGs cannot be evaluated in detail, but the resolution can be enhanced by using SEM to visualize positions of NWs (Figure 4b–d). As it is difficult to quantify exactly the geometrical parameters related to the mutual orientations of NWs and their space distribution, we just give some typical examples collected from different places and taken at different (both low and high) magnifications showing that the orientations are random at all magnification levels.

However, one should take into account that the preparation of samples for SEM includes elimination of water from FGs, which necessarily results in substantial contraction of the sample. Therefore, the positions of Ni NWs in the dry specimen prepared for SEM studies are not the same as the positions of Ni NWs in the intact FG. However, we expect that qualitative features of NWs arrangement would be preserved. Figure 4b–d shows SEM microphotographs of aggregates of NWs in a dry FG1 slice. Our previous studies indicate that sample preparation method (including the nanoscale-thick carbon layer deposition) does not change the structure of the surface layer but makes the compositional analysis very difficult as the surface become enriched by the carbon [20,21]. In any case, EDS analysis did not show other metallic elements except nickel.

Both the length and the width of NWs are clearly resolved. It is noticeable that in general NWs are bent with straight segments several microns in length. Since the NWs in optical images of the suspension (Figure 1) and of the FG (Figure 2) look straight, the observed bending might be the result of the contraction of the FG matrix during its drying. Overall, the homogeneity and morphology of the NWs is well preserved after embedding in the FG.

**Figure 5:**
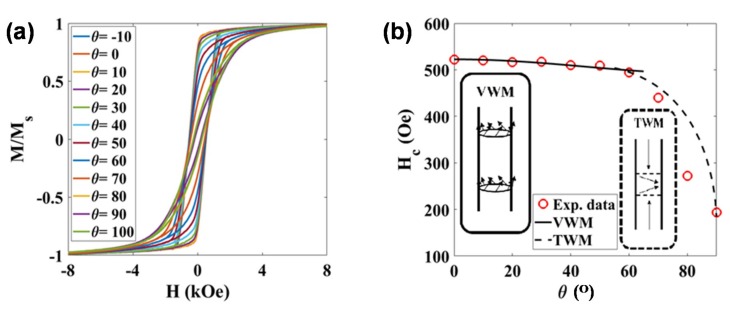
(**a**) M-H hysteresis loops measured at room temperature for the Ni nanowires inside the AAO template at different angles, *θ*, with respect to the applied field. (**b**) Evolution of the coercive field, *H*_C_, as a function of the angle, *θ*. The continuous line (0–60°) is the calculated angular dependence of *H*_C_ for the vortex domain wall motion (VWM), while the dashed line (60–90°) is a guide-for-the-eye line suggesting a transverse domain wall motion (TWM).

The magnetic response of these NWs inside and outside the hydrogel was also studied. The static hysteresis loops of the NWs in an AAO template with the nanowires oriented at different angles with respect to the magnetic field are shown in Figure 5a. In all configurations, a saturation magnetization, *M*_S_, close to 46 emu/g was obtained. This value is slightly smaller than the one expected for bulk Ni (fcc): 55 emu/g at room temperature [26]. This reduction in *M*_S_ could be related to some Ni oxidation on the surface of the nanowires, as commented before. The shape of the M-H loops is clearly different depending on the orientation. The squared shape and high remanence obtained for the M-H hysteresis loop in the parallel orientation, and the narrow and highly tilted shape of the M-H loop in the perpendicular configuration, indicate that there is a preferential magnetization direction (effective anisotropy) along the long axis of these NWs. In other words, the high aspect ratio of the NWs gives rise to a preferential shape anisotropy along the cylinder axis. Similar highly anisotropic behaviors have been reported before in other metallic NWs [26,27,28].

High aspect ratio magnetic nanowires typically present a magnetic response, as a function of the applied magnetic field, that can be interpreted as either field-driven domain wall motion or coherent rotation [29]. In order to shed some light on the dominant magnetization reversal mechanism in our NWs, we have studied the angular dependence of the coercivity, as described in [30,31]. As can be observed in Figure 5b, the coercive field, *H*_C_, reaches a maximum value of ~520 Oe in the parallel configuration (*θ* = 0°), and keeps around the same value with an increasing angle up to 60°. For higher angles, the coercive field progressively decreases down to ~200 Oe in the perpendicular configuration (*θ* = 90°). Similar values and evolution of the coercive field have been reported for Ni NWs arrays in the literature [30,31]. We have tried to fit our experimental data using the models described in Reference [30]. As indicated in Figure 5b, relatively good fittings showed by the continuous black line have been obtained for angles 0–60° considering a vortex domain wall motion model. The strength of the linear association between variables under consideration can be quantified by the correlation coefficient; the square of the correlation coefficient, a useful value in linear regression, was here very close to 0.990. The fitting parameters are the exchange stiffness constant, A, and the magnetocrystalline anisotropy constant, *K*_mc_, and the obtained values are 1.75 × 10^−6^ erg/cm and 3.96 × 10^4^ erg/cm^3^, respectively, which are very close to theoretical values for Ni [32,33]. Above 60°, the evolution of the coercive field could not be fitted with any of the proposed models, although the shape of the experimental curve seems to resemble a transverse domain wall magnetization reversal mode, as indicated with a guide-for-the-eye dashed line included in Figure 5b.

**Figure 6:**
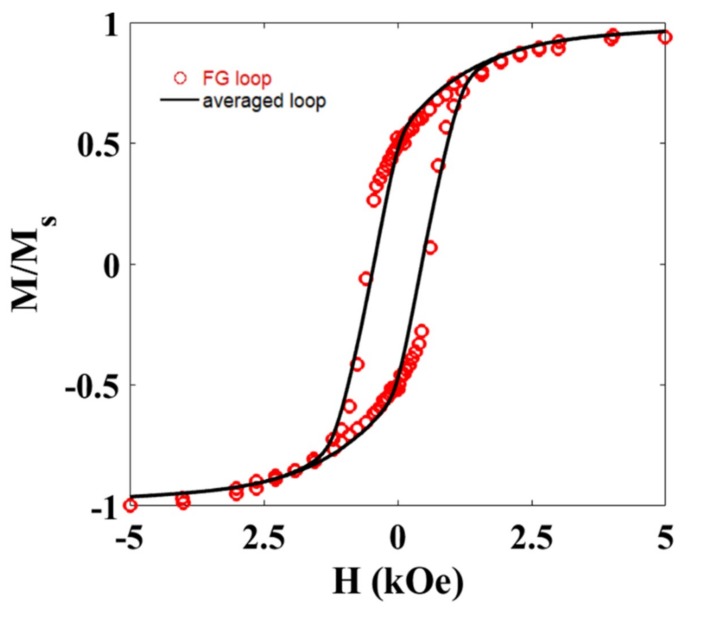
M-H hysteresis loop (FG loop) measured at room temperature for the Ni NWs dispersed in random orientations inside the FG1 sample. The results are compared with the hysteresis loop (averaged loop) obtained from averaging all the M-H loops in Figure 5a.

The magnetic response of the gel and each FG sample has also been recorded at room temperature. Figure 6 shows the M-H loop for the FG1 sample. Similar behavior was observed for the FG2 sample. 

The diamagnetic contribution from the hydrogel has been subtracted from this measurement. As observed, the squareness of the hysteresis loop is intermediate between the parallel and perpendicular configurations analyzed before, pointing towards a global random alignment of the NWs inside the hydrogel, as already suggested by microscopy images. This is further supported if we compare this M-H loop with that obtained from averaging the M-H loops measured at different angles (Figure 5a). As depicted in Figure 6, both M-H loops are very similar. Slight differences in the coercive field of both hysteresis loops could be related to the effect of dipolar interactions. 

Further work will be carried out to test the performance of these FGs in order to create multifunctional devices for applications such as regenerative medicine and magnetic biosensing. Simple prototypes of compact analytical devices working on the principle of the detection of stray fields created by iron oxide magnetic nanoparticles were designed and tested for the case of polyacrylamide-based ferrogels [11,13,33]. For the enhancement of magnetic biosensor sensitivity one can search for magnetic filler, which differ from superparamagnetic iron oxide nanoparticles with the reduced magnetic moment. The saturation magnetization of Ni NWs is higher in comparison with typically used iron oxide nanoparticles of about 15 nm diameter and therefore their employment may insure enhanced sensitivity. Encapsulation into hydrogel provides a certain degree of compatibility. For example, one can imagine a hypothetical regenerative medicine case with a magnetic field sensor based on a multilayered sensitive element for the desired point intravenous delivery of a gel-based scaffold [34].

## 4. Conclusions

Long Ni NWs with a high aspect ratio (~250) were fabricated by electrodeposition into commercial AAO templates. For the evaluation of the distribution of Ni NWs in a water suspension obtained by dynamic light scattering, the difference of translational mobility of spherical particles and particles having an ellipsoid shape was taken into account. Ni NWs exhibit a relatively high saturation magnetization (46 emu/g), and a well-defined uniaxial anisotropy as revealed by the evolution of the magnetic response as a function of the angle between the magnetic field and the NWs. A vortex domain wall mode seems to be preferred for the reversal of the magnetization inside these NWs.

Long Ni NWs were used as a magnetic filler for FGs synthesis. FGs were fabricated by free radical polymerization of monomeric acrylamide. Obtained materials were homogeneous at the mesoscopic scale, and the morphology of the NWs was well preserved after embedding in the gel. The weight fractions of magnetic filler in the FGs were either 0.036% or 0.169% by weight. Both samples present a similar magnetic response, confirming the homogeneous distribution of the nanowires inside the FG, and the random orientation of the NWs. The magnetic response of the NWs was preserved after embedding in the FG.
